# The Use of Social Media in Recruitment for Medical Research Studies: A Scoping Review

**DOI:** 10.2196/jmir.5698

**Published:** 2016-11-07

**Authors:** Jane Topolovec-Vranic, Karthik Natarajan

**Affiliations:** ^1^ Li Ka Shing Knowledge Institute St Michael's Hospital Toronto, ON Canada; ^2^ Department of Occupational Science and Occupational Therapy University of Toronto Toronto, ON Canada

**Keywords:** patient selection, social media, social networking, intervention study, observational study, Internet

## Abstract

**Background:**

Recruiting an adequate number of participants into medical research studies is challenging for many researchers. Over the past 10 years, the use of social media websites has increased in the general population. Consequently, social media websites are a new, powerful method for recruiting participants into such studies.

**Objective:**

The objective was to answer the following questions: (1) Is the use of social media more effective at research participant recruitment than traditional methods? (2) Does social media recruit a sample of research participants comparable to that recruited via other methods? (3) Is social media more cost-effective at research participant recruitment than traditional methods?

**Methods:**

Using the MEDLINE, PsycINFO, and EMBASE databases, all medical research studies that used social media and at least one other method for recruitment were identified. These studies were then categorized as either interventional studies or observational studies. For each study, the effectiveness of recruitment, demographic characteristics of the participants, and cost-effectiveness of recruitment using social media were evaluated and compared with that of the other methods used. The social media sites used in recruitment were identified, and if a study stated that the target population was “difficult to reach” as identified by the authors of the study, this was noted.

**Results:**

Out of 30 studies, 12 found social media to be the most effective recruitment method, 15 did not, and 3 found social media to be equally effective as another recruitment method. Of the 12 studies that found social media to be the best recruitment method, 8 were observational studies while 4 were interventional studies. Of the 15 studies that did not find social media to be the best recruitment method, 7 were interventional studies while 8 were observational studies. In total, 8 studies stated that the target population was “hard-to-reach,” and 6 of these studies found social media to be the most effective recruitment method. Out of 14 studies that reported demographic data for participants, 2 studies found that social media recruited a sample comparable to that recruited via traditional methods and 12 did not. Out of 13 studies that reported cost-effectiveness, 5 studies found social media to be the most cost-effective recruitment method, 7 did not, and 1 study found social media equally cost-effective as compared with other methods.

**Conclusions:**

Only 12 studies out of 30 found social media to be the most effective recruitment method. There is evidence that social media can be the best recruitment method for hard-to-reach populations and observational studies. With only 30 studies having compared recruitment through social media with other methods, more studies need to be done that report the effectiveness of recruitment for each strategy, demographics of participants recruited, and cost-effectiveness of each method.

## Introduction

For any study, recruitment of an adequate number of participants who reflect the targeted population is essential. Failure to achieve this goal can compromise the validity of the results, increase costs, and delay or even cause early termination of the study [1]. This is a major problem today; less than 20% of clinical trials finish on time [2]. Roughly half of these delays are due to difficulties in patient recruitment [2].

Web 2.0, or interactive communication through the Web represents a valuable method of sharing information. In 2015, 90% of Canadian households had access to the Web [3]. At the forefront of Web usage today are social media websites. For the purposes of this paper, social media websites are defined as websites that let users make profiles and use these profiles to connect and interact with other individuals. The use of such websites is constantly growing, reflecting the population as a whole. As of 2015, the majority of Canadians use social media. The most popular social media website is Facebook with 59% of Canadians having an account [4]. While detailed statistics on the increasing use of social media are not available for Canada, in the United States, 65% of US adults used a social media website in 2015, an increase from 7% in 2005 [5]. While use increased from 12% to 90% from 2005 to 2015 for the age group 18-29 years, more recently its use has increased rapidly in older populations—it is now used by 77% of 30- to 49-year-olds, 51% of 50- to 64-year-olds, and 35% of those aged 65+ years, increasing from 8%, 5%, and 2%, respectively, in 2005 [5]. Furthermore, 56% of low-income individuals now report using social media [5].

As a result of these increases in social media usage over the last few years, social media represents a potential source for recruitment of participants. Studies have shown that a high volume of individuals can be successfully recruited for research purposes using social media [6-8]. Researchers have utilized these sites, such as Facebook, for recruitment of individuals into their studies [6,7]. Recruitment through this method has been shown to be cost-effective [6-8]. Additionally, social media has been shown to recruit populations that cannot be easily accessed through traditional methods of recruitment [9,10], a specific example of which is low-income populations [11].

Literature reviews on the role of social media in recruitment have been done by Park and Calamaro [12] and Ryan [13]. These reviews also identified social media as being effective in recruiting both hard-to-reach populations and adolescents and young adults (AYAs), as well as being cost-effective. However, the majority of these studies have only looked at AYAs and not older populations where social media usage has increased. Furthermore, many of these studies have not directly compared recruitment via social media with that via traditional methods. To fill these gaps, a scoping review was conducted to answer the following questions: (1) Is social media more effective at research participant recruitment than traditional methods? (2) Does social media recruit a sample of research participants comparable to that recruited via other methods? (3) Is social media more cost-effective at research participant recruitment than traditional methods?

## Methods

### Search Strategy

A scoping review was performed using the Preferred Reporting Items for Systematic Reviews and Meta-Analyses (PRISMA) guidelines. Articles appearing in a journal and written in English were included. Review articles, abstracts, dissertations, narratives, and letters were excluded.

#### Types of Participants

Study participants included adults and children participating in any health care–related research study including recruitment via social media.

#### Types of Interventions

Any type of interventional study or observational study was included.

#### Types of Controls or Comparators

Studies with recruitment via at least one other method such as newspaper, in person, and telephone were included.

#### Types of Outcomes

Three outcomes were assessed for this review: (1) *effectiveness* of recruitment, (2) *comparativeness* of recruited participants in relation to the population of interest, and (3) *cost-effectiveness* of recruitment. The *effectiveness* of recruitment was measured as the number of participants recruited via social media over a given time period as compared with the other recruitment methods. *Comparativeness* of the recruitment of participants was assessed by comparing the demographic characteristics of patients recruited via social media with that of other methods. *Cost-effectiveness* of each recruitment method was determined by dividing the total cost of advertisement for a particular recruitment strategy by the total number of participants recruited through that strategy.

#### Information Sources and Search Strategy

The original literature search was conducted between July 8 and July 11, 2014, using the databases MEDLINE (1946-2014), PsycINFO (1987-2014), and EMBASE (1980-2014 week 27). The search was updated on May 7 and May 8, 2015, as well as on July 26 and July 27, 2016. The search terms for the MEDLINE database were as follows: (“Recruit*” OR “Patient Selection (MeSH) (Medical Subject Headings) or Patient Recruit*” OR “Subject Recruit*” OR “Participant Recruit*” OR “Recruit* Strategies”) AND (“Social Media (MeSH) or Social Media” OR “Social Network” OR “Social Networking (MeSH) or Social Networking” OR “Facebook” OR “Youtube” OR “Qzone” OR “Sina Weibo” OR “WhatsApp” OR “Google+” OR “Tumblr” OR “Twitter” OR “WeChat” OR “Tencent Weibo” OR “LinkedIn” OR “Youku” OR “Instagram” OR “Tudou” OR “RenRen” OR “Pinterest” OR “Badoo” OR “Orkut” OR “Foursquare” OR “Vine” OR “Vkontakte” OR “Myspace” OR “Snapchat” OR “Reddit” OR “Bebo” OR “Hi5”). Multimedia Appendix 1 contains the full search strategy. After the articles were found, the reference lists of relevant studies were searched for additional studies. To be as comprehensive as possible, social media sites used primarily outside North America were also included in the search.

### Screening Process

The screening process involved 2 stages: (1) title and abstract exclusion and (2) full-text exclusion. Titles were excluded if they were not related to health care or the topic of social media and recruitment. Abstracts were excluded if they were not a primary journal article, unrelated to social media and recruitment, or did not use social media in the recruitment strategy. Full-text studies were excluded if they did not measure the primary outcome (*effectiveness* of recruitment) or did not have an appropriate control group.

### Data Extraction

The relevant studies were then screened for data, including the number of people recruited via each method, the demographic characteristics of the study participants (age, sex, ethnicity, economic status, and educational level), characteristics of the study (country of origin, social media sites used, other recruitment methods, the method used to measure primary outcome, and geographic distribution), reported costs of recruitment activities, and incentives.

## Results

### Study Selection

**Figure 1:**
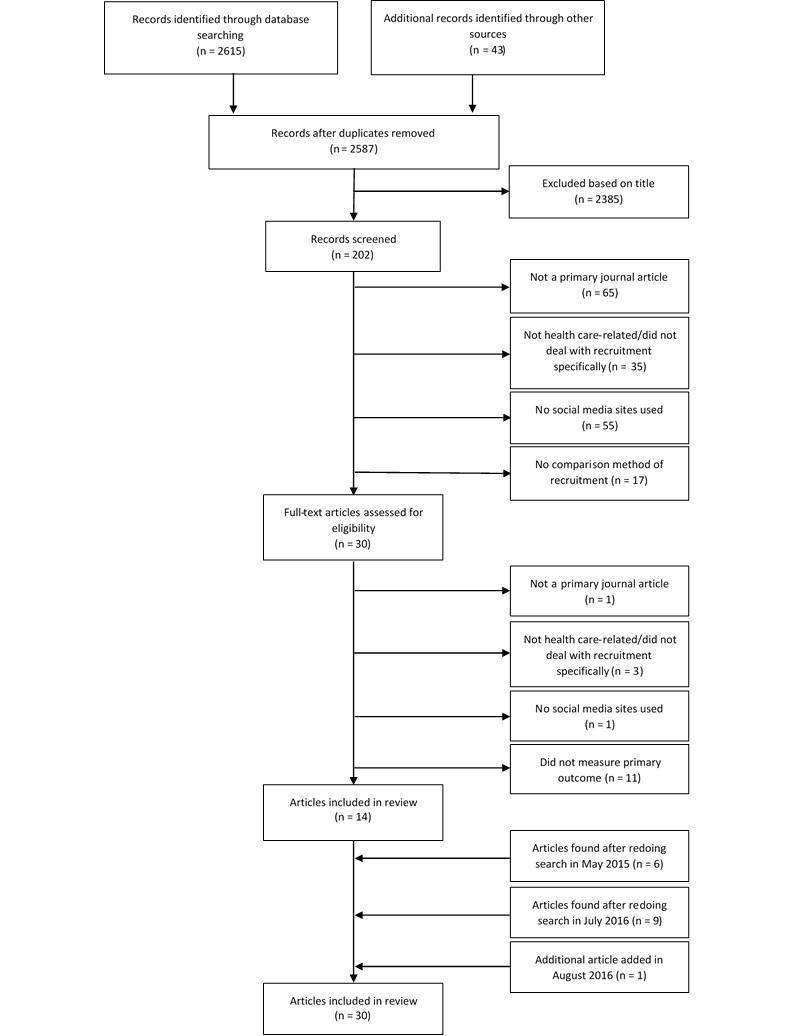
Search strategy results.

The search produced 2658 results, out of which 71 results were duplicates (Figure 1), leaving 2587 results. From these results, 2385 were excluded because the titles were irrelevant to the topic of social media and recruitment, leaving 202 abstracts to be reviewed. From this, 172 more abstracts were excluded because they were not primary research articles (n=65), were not health care–related or did not deal with recruitment specifically (n=35), did not use social media for recruitment (n=55), or did not have a comparison recruitment method (n=17). This left 30 full-text articles to be assessed for eligibility. Out of this total, 16 more of these studies were excluded because they did not measure the number of people recruited via social media over a given period of time (n=11), were not health care–related (n=3), did not use social media sites (n=1), or were not primary research articles (n=1). A total of 6 additional studies were found after redoing the search in May 2015, 9 additional studies were found in July 2016, and 1 additional study was added in August 2016, for a total of n=30 articles that were included in the review.

### Recruitment Effectiveness

The percentage of participants recruited via social media ranged from 0% (0/12) to 98.29% (1610/1638) [14-42] as shown in Table 1, and the median percentage was 32%. The article by Head et al [35] has 2 studies and has been counted as 2 articles for the purpose of Figure 1. In further sections of this paper, the article by Head et al [35] is counted as a single article or 2 articles, according to whether the conclusions from the 2 studies pertinent to the outcomes of this paper are the same or different. Out of 30 studies, 12 studies (40%) reported higher rates of recruitment through social media as compared with any of the other methods used [14-17,26,28,31,32,35, 36,41,42] and 15 studies (50%) reported recruitment via social media to be less effective than at least one other method used [18-21,23-25, 27,33-35,37-40]. Heffner et al [20] and Rabin et al [24] found social media to be the least effective method out of multiple (>2) recruitment methods used. Rabin et al [24] were unable to recruit a single participant via social media.

**Table 1:** The percentage of participants recruited through social media by study (the number of participants recruited through social media is also provided in parentheses, when reported).

Primary article	Percentage of participants recruited through social media
Balfe et al [14]	76% (29/38)
Frandsen et al [15]	51.9% (138/266)
Johnson et al [16]	49.6% (402/811)
Yuan et al [17]	81.09% (1544/1904)
Burrell et al [18]	23.8% (24/105)
Graham et al [19]	8.0% (40/500)
Heffner et al [20]	5.0% (11/222)
Layi et al [21]	20.0%
Martinez et al [22]	36% (5/14)
Quach et al [23]	17.0% (81/477)
Rabin et al [24]	0% (0/12)
Shere et al [25]	4% (2/45)
Theriault et al [26]	83.8% (201/240)
Vial et al [27]	12.77% (163/1276)
Carlini et al [28]	41.4% (286/690)
Haines-Saah et al [29]	28% (17/60)
Miyagi et al [30]	52.3% (127/243)
Wilkerson et al [31]	93.3% (320/343)
Ince et al [32]	77% (74/96)
Hernandez-Romieu et al [33]	13.7% (110/803)
Rait et al [34]	22.5% (45/200)
Head et al [35], study 1	98.29% (1610/1638)
Head et al [35], study 2	3.8% (5/131)
Kayrouz et al [36]	86% (70/81)
Gu et al [37]	37.4% (58/155)
Subbaraman et al [38]	7.0%
Khatri et al [39]	18.2% (96/527)
Partridge et al [40]	2.0% (5/250)
Carter-Harris et al [41]	91.7% (331/361)
Frandsen et al [42]	52.6% (92/175)

Of the 12 studies that found social media to be the best method of recruitment, 8 were observational studies [14,16,17,26,28,31,35,41] and the remaining 4 were interventional studies [15,32,36,42], as shown in Multimedia Appendix 2. In addition, 6 of these studies targeted populations deemed hard to reach [16,17,28,31,32,36], and 6 studies targeted specific conditions or disorders [14,16,17,28,31,32]. Furthermore, 1 study targeted only young adults (aged 23-30 years) [14]. Among the 12 studies, 8 studies used only Facebook for recruitment [14-16,28,35,36,41,42]. Of the remaining 4 studies, 2 studies used a combination of Facebook and Twitter [31,32] and 2 studies used a combination of Facebook and other social media websites [17,26].

Of the 15 studies that did not find social media to be the best method, 7 studies were interventional studies [18-21,24,25,40], whereas 8 were observational studies [23,27,33-35,37-39]. Of these studies, 3 studies specifically targeted young and middle-aged adults [24,25,40], 2 studies targeted adolescents [34,37], and 2 studies targeted older adults [35,41]. Martinez et al [22] recruited 35.7% of participants via social media and 35.7% of participants via community-based organizations. Haines-Saah et al [29] recruited 28% of participants via social media and 28% of participants via friend referral. Miyagi et al [30] recruited 52% of participants through Facebook and 48% via a website. The studies by Martinez et al [22] and Haines-Saah et al [29] were both interventional studies, whereas the study by Miyagi et al [30] was an observational study.

### Demographics

A total of 23 studies reported the geographic regions targeted by social media during recruitment, as shown in Multimedia Appendix 2. Among these, 13 studies targeted local regions within a country [15,18,22,23,26,27,30,33-35,37,40,42], 8 studies [14,19,28,31,35,38,39,41] targeted recruitment nationally, and 2 studies targeted recruitment internationally [16,36].

Only 13 studies out of 30 reported at least one demographic characteristic for patients recruited through social media and other methods [15-18,20,25,28,33,35,37,41,42], with 10 studies providing in-depth demographic information [15,16,20,25,33,35,37,41,42]. However, Shere et al [25] and Yuan et al [17] included Craigslist in their definition of social media; therefore, their demographic analysis was not included in this review because Craigslist does not fall under our definition of a social media website.

With respect to ethnicity, it was found that there was no significant difference between recruitment strategies in 5 studies [15,16,20,35,37]. Despite social media recruiting different percentages of white and black participants compared with other avenues, Hernandez-Romieu et al [33] concluded that social media did not have a racial bias in recruitment, as in this case the researchers were deliberately aiming at a 50% white and 50% black sample. However, Burrell et al [18], who used Grindr to recruit, noted a significantly increased white population when compared with traditional methods, which they attributed to the fact that Grindr could only be used by individuals possessing a smartphone. Head et al [35] (studies 1 and 2) and Carter-Harris et al [41] also noted a significantly increased white population recruited through Facebook. Out of the 10 studies that formally measured the age of participants recruited, 3 recruited a comparable sample [16,28,41]. There was a younger median age in 3 studies [15,20,42], and 1 study [18] had a much higher proportion of 18- to 30-year-olds recruited via social media (56% vs 18.8%). Quach et al [23], while not formally reporting demographics, noted that social media recruited younger individuals. Although not included in the demographic analysis, Yuan et al [17] also noted that the proportion of individuals aged 60+ years recruited through Facebook was lower than that for other age groups. Hernandez-Romieu et al [33], on the other hand, noted that participants recruited via Facebook were typically older than those recruited via other avenues, and this difference was significant for black participants recruited. Head et al [35] also noted an older median age in studies 1 and 2, which was attributed to the fact that Craigslist, the main comparative recruitment method used, is more popular with younger individuals. Out of the 8 studies that reported the sex of recruited participants [15,16,20,28,35,37,41,42], 7 studies recruited a comparable sample through social media [15,16,20,28,35,41,42]. The economic status of individuals was reported in 6 studies and no significant differences were found [15,33,35,41,42]. However, Balfe et al [14] noted that social media recruited more middle-class individuals. A total of 7 studies provided information about educational attainment of recruited individuals [15,18,20,33,35,41]. It was found that education levels were higher in the social media group than in the traditional media group in 2 cases [18,20], and Hernandez-Romieu et al [33] found this to be the case for white participants recruited. Head et al [35] (study 1) noted lower education levels for individuals in the social media group, which was attributed to the fact that Craigslist is more popular with better educated individuals. Quach et al [23] also noted that education levels were higher in the social media recruitment group.

### Cost-Effectiveness and Incentives

A total of 13 studies directly compared cost-effectiveness across different recruitment strategies [15,16,19,20,28,31,33,34,36,37,39,41,42], and the results are presented in Table 2. The cost of advertisement on social media websites was determined by bidding prices for ads, which varied on a daily basis, or the cost of placing a banner ad on a particular website. Among these studies, 5 studies [16,31,33,36,41] found social media to be the most cost-effective method, whereas 7 studies found it less cost-effective than another method used [15,19,20,28,34,37,42]. Wilkerson et al [31] reported no cost using social media for recruitment, and Khatri et al [39] reported no costs for all methods used. Among the 5 studies that found social media to be the most cost-effective method, 4 were observational studies [16,31,33,41], whereas 1 study was an interventional study [36]. Of the 7 studies that found recruitment through social media less cost-effective than another method, 4 were interventional studies [15,19,20,42] and 3 were observational studies [28,34,37]. Despite not formally measuring cost-effectiveness, Theriault et al [26] noted social media to be “less costly” than traditional methods. This study was also an observational study. A total of 15 studies reported the use of incentives during recruitment, 12 of which were monetary [14,15,22,23,25,29-31,34,35,41] and 1 of which was nonmonetary [17]. The remaining 2 studies used a combination of monetary and nonmonetary incentives [37,40]. Quach et al [23] specifically looked at the effect of incentives on recruitment. Recruitment was split into 2 phases: phase 1, which offered a Can $5 gift card upon survey completion, and phase 2, which had no incentives. It was found that phase 1 attracted significantly more individuals than phase 2 (355 vs 125).

**Table 2:** Cost of recruitment for different strategies.

Reference	Cost of recruitment
Frandsen et al [15]	Facebook, AU $42.34/participant; newspaper, AU $21.52/participant.
Johnson et al [16]	First ad campaign: Google, US $13.43/registrant; Facebook, US $3.80/registrant. Second ad campaign: Google, US $18.97/registrant; Facebook, US $7.71/registrant. Mailed campaign, US $154.95/registrant.
Graham et al [19]	Myspace Latino, US $600/registrant; Yahoo en Espanol, US $119.95/registrant; MSN Latino, US $141.15/registrant; MiGente, US $4166.67/registrant.
Heffner et al [20]	Social media, US $172.76/participant; standard media, US $46.98/participant; broadcast emails, US $27.10/participant; word of mouth, US $5.27/participant; medical Internet media, US $26.19/participant; Google AdWords, US $50.26/participant.
Carlini et al [28]	Facebook, US $8.92/respondent. Google, US $16.22/respondent. Email, US $5.95/respondent. Newsletter: Florida, US $13.12/respondent; New Jersey, US $35.60/respondent; California, US $250.00/respondent.
Wilkerson et al [31]	Facebook, US $0/participant; email, US $0/participant; mobile ads, US $375.00/participant; browser ads, US $187.50/participant.
Hernandez-Romieu et al [33]	Facebook, US $68.6/participant; venues, US $91.2/participant.
Rait et al [34]	Facebook, US $149.64/registrant; bus ads, US $255/registrant; referral, US $7/registrant; school talks, US $336/registrant; fliers, US $10/registrant.
Kayrouz et al [36]	Facebook, US $37/participant; traditional, US $40/participant.
Gu et al [37]	Facebook, US $30.29/participant; Twitter, US $22.20/participant; QR Codes, US $6.57/participant.
Khatri et al [39]	US $0 for all methods.
Carter-Harris et al [41]	Facebook, US $1.51/participant; newspaper, US $40.80/participant.
Frandsen et al [42]	Facebook, AU $56.34/participant; traditional media, AU $52.33/participant.

### Setting

Out of 30 studies, 18 studies were done in the United States [16-22,24,27,28,31,33-35,37,38,41], 5 studies in Australia [15,26,36,40,42], 3 studies in Canada [23,25,29], 1 study in Ireland [14], 1 study in the Netherlands [32], 1 study in Japan [30], and 1 study in the United Kingdom [39].

**Figure 2:**
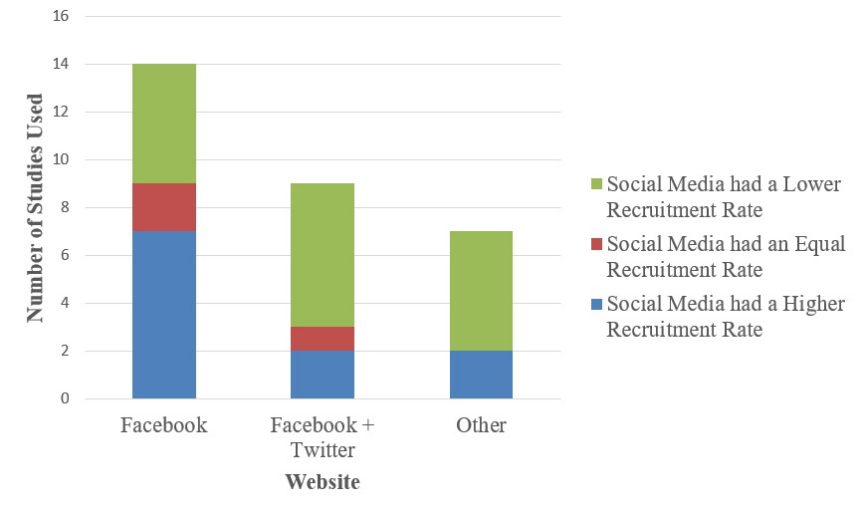
Recruitment success based on social media website(s) used. Note: Other = GRINDR, Myspace Latino + MiGente, Facebook + Google AdWords, Facebook + Twitter + Ning, Facebook + Gaydar, Facebook + Twitter + Youtube, Facebook + Twitter + LinkedIn + Tumblr, Facebook + Twitter + Instagram + Grindr + Jack’d + Scruff.

It was found that 14 out of 30 studies used Facebook solely [14-16,27,28,30,33-36,40-42]. Although Twitter was never used by itself, 9 studies used a combination of both Facebook and Twitter during the recruitment process [20,21,23,25,29,31,32,37,38]. The overwhelming majority of studies (28/30) used Facebook in some way during recruitment, indicating that this was the most popular website for this purpose. The only times Facebook was not used were when there were social media sites that targeted a specific population of interest, such as MSM (men who have sex with men) [18] and Latinos [19]. Other recruitment methods included visiting various community venues such as clubs and bars, health care centers, and universities. There was also recruitment done via numerous websites that would not be classified as social media based on our definition, such as Craigslist, Kijiji, and Google AdWords. Overall, using a combination of social media websites, or not using Facebook, resulted in lower recruitment through social media (Figure 2).

## Discussion

### Principal Findings

It was found that social media was the most effective method in 12/30 studies and not the most effective method for recruiting patients in 15/30 studies. The effectiveness of social media for recruitment of study participants is highly variable and dependent on specific study characteristics such as age, whether the population is difficult to reach through traditional methods, and the method used to measure the primary outcome. This contrasts with the finding that social media is a highly effective recruitment method presented in studies such as those by Fenner et al [6] and Ramo and Prochaska [7]. One possible reason for this is the fact that these studies did not use other methods of recruitment, and therefore solely focused their efforts on recruitment through social media. Among the studies where the effort put into each recruitment method was discernible, it was generally found that the effort put into recruitment via social media correlated with the number of participants recruited through this method. Effort was defined as the combination of the number of social media websites used, the extensiveness of the social media recruitment strategy as compared with that of traditional methods, the frequency with which recruitment was conducted, and the time spent on recruitment through social media when this information was reported. Studies that put more effort into recruitment via social media than via other methods generally recruited the most number of participants through social media [17,22,32,36,41] and vice versa [18,20,25,27,33,37,38,40].

It was found that in 2 cases [16,28] social media was able to recruit a sample that was comparable to the control group. However, in 12 cases the sample recruited via social media was not comparable to the general population. Participants were found to be younger [15,17,18,20,23,42], older [33,35], more white [18,35,41], had a higher education level [18,20,23,33], had a lower education level [35], more female [37], and had higher socioeconomic status [14]. It was also noted that all studies were from developed countries.

There is evidence that social media is best able to recruit individuals for observational survey-type studies as opposed to interventional studies; however, with a limited number of studies (n=12) to evaluate, more studies are needed. There is also evidence that social media can be a better recruitment method than other Internet sources alone. Of the 7 studies that compared recruitment via social media only with other Internet sources, 5 found social media to be the top method of recruitment [17,28,30,31,35]. Studies that targeted more specific groups, rather than a more general audience, can also potentially be more successful at recruiting via social media. For instance, social media seemed to be successful at recruiting hard-to-reach populations [16,17,28,31,32,36] and individuals with specific conditions or disorders [14,16,17,28,31,32]. This finding was in agreement with the findings of Park and Calamaro [12] and Ryan [13]. This is likely because in such a case it is difficult for any one conventional source to find a sufficient number of individuals, as was noted in the study by Johnson et al [16]. Once again, however, there is limited evidence for this. More studies need to be done looking at the effectiveness of recruitment using social media in these specific groups. Interestingly, the use of multiple social media websites appeared to result in lower recruitment through social media. When multiple social media websites were used, however, the most successful website at recruitment was Facebook. Low recruitment through Facebook alone typically indicated low overall recruitment through social media and vice versa. Therefore, we speculate that this finding is not due to the use of multiple social media sites but due to the success of recruitment through other methods in these studies.

A total of 5 studies found social media to be the most cost-effective method [16,31,33,36,41], whereas 7 studies found that it was not the most cost-effective method [15,19,20,28,34,37,42]. Therefore, no significant conclusions on cost-effectiveness can be made. This finding is slightly different from Park and Calamaro’s [12] conclusion of social media being cost-effective. One potential explanation for this is that other studies, which focused solely on recruitment using social media, recruited higher numbers of individuals (450 in the study by Ramo and Prochaska and 426 in the study by Fenner et al) [6,7]. However, these studies also did not compare cost-effectiveness of other methods in recruiting the same target population, so it is uncertain whether traditional methods would be even more cost-effective in these cases. Additionally, the sample sizes of both the study by Park and Calamaro [12] (n=3) and our review (n=12) are likely too small to draw highly accurate conclusions. The cost of recruitment is also highly variable and depends on interactions between recruitment sites, study size, and target population. Advertisements are additionally affected by the bid price needed to display the advertisements, as noted by Fenner et al [6] and Ramo and Prochaska [7]. Therefore, a more complex analysis is needed to understand cost-effectiveness when recruiting through social media.

Recruitment through social media is affected by several factors. Quach et al [23] explicitly showed that adding a monetary incentive can increase recruitment through social media. Although 1 study represents limited evidence for the effectiveness of incentives, this finding is in line with the conclusion by Bower et al [43] that monetary incentives can increase recruitment into medical health studies. Another important factor is sex. It has been shown that women are more likely to search the Web for health information than men [44] and are more likely to participate in health studies [45]. Although no differences between male and female recruitment were found in this review, having an adequate representativeness in sex needs to be kept in mind by researchers when designing recruitment mechanisms.

When recruiting a target population, it is also important to consider how that population uses social media. For instance, for young MSM, Holloway et al [46] noted that this population was more likely to use dating sites when meeting new sexual partners and used Facebook when communicating with individuals they already knew. Therefore, researchers interested in targeting this population for a sexual health study should use these dating sites for recruitment and use Facebook for a nonsexual health study. Some social media sites are also more popular among certain demographics—for instance, within MSM, Grindr is more popular among whites, whereas Jack’d is more popular among African Americans [47].

Overall, researchers should consider how the target population uses social media when deciding which recruitment strategies to use, taking into account factors such as age, sex, the likelihood of a comparable sample, and whether the population would be difficult to reach through traditional methods. Even if social media can recruit more individuals than other methods, researchers must still estimate the cost-effectiveness of recruitment via this method, and in the event that cost-effectiveness is low, determine if recruitment is worth the low cost-effectiveness.

### Limitations of Using Social Media for Research Recruitment

Ads on social media websites were targeted at specific age groups and locations based only on the information an individual provided on his or her profile. Therefore, there is no guarantee that awareness of the study reached all potential participants, and this could bias the results. Many studies created a separate page to recruit participants. Once again, not all potential participants may have been made aware of this page. For the studies that involved surveys, individuals could have reported false demographic information in the survey or could have given multiple responses, and verification of information on the Web remains more difficult than in person. In addition, individuals may not have correctly reported their source of recruitment, as Johnson et al [16] noted.

Within social media itself different types of recruiting strategies were used across different studies, such as creating a separate page to advertise the study, targeted advertisements, and private messages. Different strategies can alter the number or demographics of participants recruited and thus may not necessarily lead to a fair comparison between social media and other methods.

There is also the possibility that neither social media nor traditional methods were representative of the target population, as Ince et al [32] and Gu et al [37] noted. This can result from self-selection bias, where individuals who agree to participate in a study are more motivated than the general target population, and the demographic characteristics of these individuals differ from the remainder of the target population [48]. Although this may limit the ability to have a representative outcome when recruiting with social media, if researchers can understand the ways in which self-selection bias takes place, then recruitment of a representative outcome, as compared with the target population, is still possible. For example, Fenner et al [6] noted that, at their study site, rural participants were underrepresented because of the increased driving distance to reach the site. Oversampling of rural participants can therefore create a representative outcome [6].

### Limitations of This Study

To identify relevant studies, an extensive list of keywords was used in the search strategy, and the reference lists of the identified studies were additionally scanned in order to extract more relevant studies. However, although we have tried to be as thorough as possible in identifying the literature, it is possible that some relevant studies were missed. Also, given the rapidly growing adoption of social media, we anticipate this body of literature to expand exponentially; this review is limited to studies published before August 10, 2016. Although we included all popular social media sites in the search strategy, not all existing social media sites were included because of the sheer number of such sites. Additionally, only studies written in English were selected.

This review looked at the recruitment strategies of different studies, rather than the main result of these studies themselves. To the best of our knowledge, there is no checklist for measuring the quality of recruitment strategy. Therefore, the quality of these studies cannot be measured in this regard.

Another limitation of the study is that the definition of a social media website varies across the literature. For instance, according to the definition by Shere et al [25], the sites Craigslist and Kijiji would be classified as social media, and so these authors concluded that social media was the most effective method owing to high recruitment via Craigslist and Kijiji even though recruitment via Facebook and Twitter was low. According to the definition by Theriault et al [26], the site Gaydar would not be classified as social media, but under our definition it would be. In such cases, we tried to fit the results to our definition of a social media site, but others may have a different definition of a social media site. This has an effect on the conclusions that can be drawn about recruitment of participants.

In addition, to measure the comparativeness of the population recruited, demographic characteristics of participants recruited through social media were only compared with characteristics of those recruited through other methods. We cannot rule out the possibility that neither social media nor traditional methods had outcomes that were representative of the target population, as is what occurred in the studies by Ince et al [32] and Gu et al [37]. This can limit the conclusions that can be made regarding representativeness.

### Future Directions

Despite several studies pointing to social media as a potential method of recruiting patients in the preliminary search, the fact that only 30 studies were identified that explicitly compare recruitment methods shows that more studies need to be done in this area. Furthermore, several of these studies also did not assess the demographics of the recruited participants—such as age, ethnicity, income, and education level—or the cost-effectiveness of each recruitment strategy. In order to truly assess the viability of social media as a recruitment tool, future studies should measure these factors as well. Studies that found social media to be effective tended to target specific populations and used surveys, but sample sizes were too low to make strong conclusions. More studies need to be done to determine the validity of these statements.

### Conclusions

Given the rising cost of conducting health research, and increased competition for such funds in Canada, new and innovative methods to recruit study participants are needed. Leveraging the growing popularity of social media has the potential to enhance research recruitment methods. However, based on our scoping review of the literature, social media was found to be the best recruitment method in only 12 out of 30 (40%) studies assessed in terms of number of individuals recruited. Social media also tended to recruit younger individuals (when this information was reported). However, for hard-to-reach populations, for populations with specific conditions or disorders, and for observational studies, social media can potentially be the most effective recruitment strategy. Although many studies used social media in recruitment, only 30 studies have explicitly compared social media with other recruitment methods. Additionally, many of these studies did not measure demographics of the population recruited. Therefore, more studies need to be done in this area. These studies should not only measure how many participants can be recruited through each strategy, but also clearly report demographics and the cost-effectiveness of each strategy.

## Appendix

Multimedia Appendix 1 Detailed search strategy.

Multimedia Appendix 2 Study characteristics, recruitment effectiveness by method, demographics of participants recruited by method, and geographic areas targeted by method.

## Abbreviations

AYA: adolescent and young adult
MeSH: Medical Subject Headings
MSM: men who have sex with men
PRISMA: Preferred Reporting Items for Systematic Reviews and Meta-Analyses
